# The Role of NRF2 in Bone Metabolism – Friend or Foe?

**DOI:** 10.3389/fendo.2022.813057

**Published:** 2022-02-23

**Authors:** Jie Han, Kuan Yang, Jinyang An, Na Jiang, Songbo Fu, Xulei Tang

**Affiliations:** ^1^ The First Clinical College of Lanzhou University, Lanzhou, China; ^2^ Department of Endocrinology, The First Hospital of Lanzhou University, Lanzhou, China

**Keywords:** NRF2, osteoporosis, reactive oxygen species, osteoblasts, osteoclasts

## Abstract

Bone metabolism is closely related to oxidative stress. As one of the core regulatory factors of oxidative stress, NRF2 itself and its regulation of oxidative stress are both involved in bone metabolism. NRF2 plays an important and controversial role in the regulation of bone homeostasis in osteoblasts, osteoclasts and other bone cells. The role of NRF2 in bone is complex and affected by several factors, such as its expression levels, age, sex, the presence of various physiological and pathological conditions, as well as its interaction with certains transcription factors that maintain the normal physiological function of the bone tissue. The properties of NRF2 agonists have protective effects on the survival of osteogenic cells, including osteoblasts, osteocytes and stem cells. Activation of NRF2 directly inhibits osteoclast differentiation by resisting oxidative stress. The effects of NRF2 inhibition and hyperactivation on animal skeleton are still controversial, the majority of the studies suggest that the presence of NRF2 is indispensable for the acquisition and maintenance of bone mass, as well as the protection of bone mass under various stress conditions. More studies show that hyperactivation of NRF2 may cause damage to bone formation, while moderate activation of NRF2 promotes increased bone mass. In addition, the effects of NRF2 on the bone phenotype are characterized by sexual dimorphism. The efficacy of NRF2-activated drugs for bone protection and maintenance has been verified in a large number of *in vivo* and *in vitro* studies. Additional research on the role of NRF2 in bone metabolism will provide novel targets for the etiology and treatment of osteoporosis.

## 1 Introduction

Bone, as a mineral reservoir and a highly dynamic tissue, is composed of about 10% cells, 60% mineral crystals (hydroxyapatite crystals) and 30% organic matrix (1). Calcium and phosphate can be mobilized to retain mineral homeostasis (2). The osteoblasts originating from mesenchymal stem cells are responsible for mineral deposition (3), whereas the osteoclasts derived from the hematopoietic lineage of monocytes/macrophages possess the ability to absorb mineralized matrix (4, 5). The bone metabolism is composed of bone modeling and bone remodeling. Bone modeling occurs from the fetus to bone matures and is a process in which bones change their shape in response to physiological factors or mechanical forces. Bone formation or resorption occurs unidirectionally at different locations and time periods of the bone, which is essential for the acquisition of peak bone mass (6). Bone remodeling is the process of renewing bones to maintain bone strength and mineral metabolism homeostasis throughout life. Physiologically, the cavity absorbed by osteoclasts is refilled with new bone formed by an equal number of osteoblasts, constantly repairing minor bone injuries to maintain bone mechanical strength (7, 8). Bone formation and resorption are closely coupled and regulated by various hormones, nervous system and mechanical factors during this process (1, 7, 9). The main hormone regulators in bone metabolism involve calcitonin (CT), parathyroid hormone (PTH), [1,25 (OH) vitamin D], sex hormones, thyroid hormones, glucocorticoids and growth hormones. Growth factors, such as IGF, TGF-beta, FGF, EGF, WNT and BMP also play an important role in regulating physiological bone remodeling (2, 4). The balance of bone remodeling depends on the precise coupling control of bone formation and bone resorption. The balance is impaired under pathological conditions and it leads to a variety of acquired metabolic bone diseases, including osteoporosis (1, 10).

Osteoporosis is a classic age-related bone disease characterized by low bone mineral density, deterioration of bone microstructure and subsequent increase in bone fragility. Fragility fracture is the most serious complication of osteoporosis (11). The number of multicellular bone remodeling units is significantly increased in osteoporosis patients, leading to concomitant increased bone absorption and bone formation rates. Since the velocity of bone absorption is faster than that of bone formation, bone remodeling imbalance results in persistent bone loss (12). Accumulated evidence has shown that primary osteoporosis cannot be simply attributed to aging and sex hormone deficiency. However, the increased levels of oxidative stress and inflammation may be the main cause of primary osteoporosis and oxidative stress plays a central role in the study of bone metabolism (13–15). At present, the molecular mechanism of oxidative stress in bone metabolism mainly involves FOXOs, SIRT, P53, NF-κB, MAPK, NOX, PPARgamma, WNT/beta-catenin, etc (13, 16). It is necessary to clarify the role of key molecules and signaling pathways in bone formation and resorption in order to explore the effective therapeutic intervention targets. NRF2, one of the core molecules of the oxidative stress-related signaling pathway, has attracted much attention in the studies of bone metabolism in recent years.

**Figure 1:**
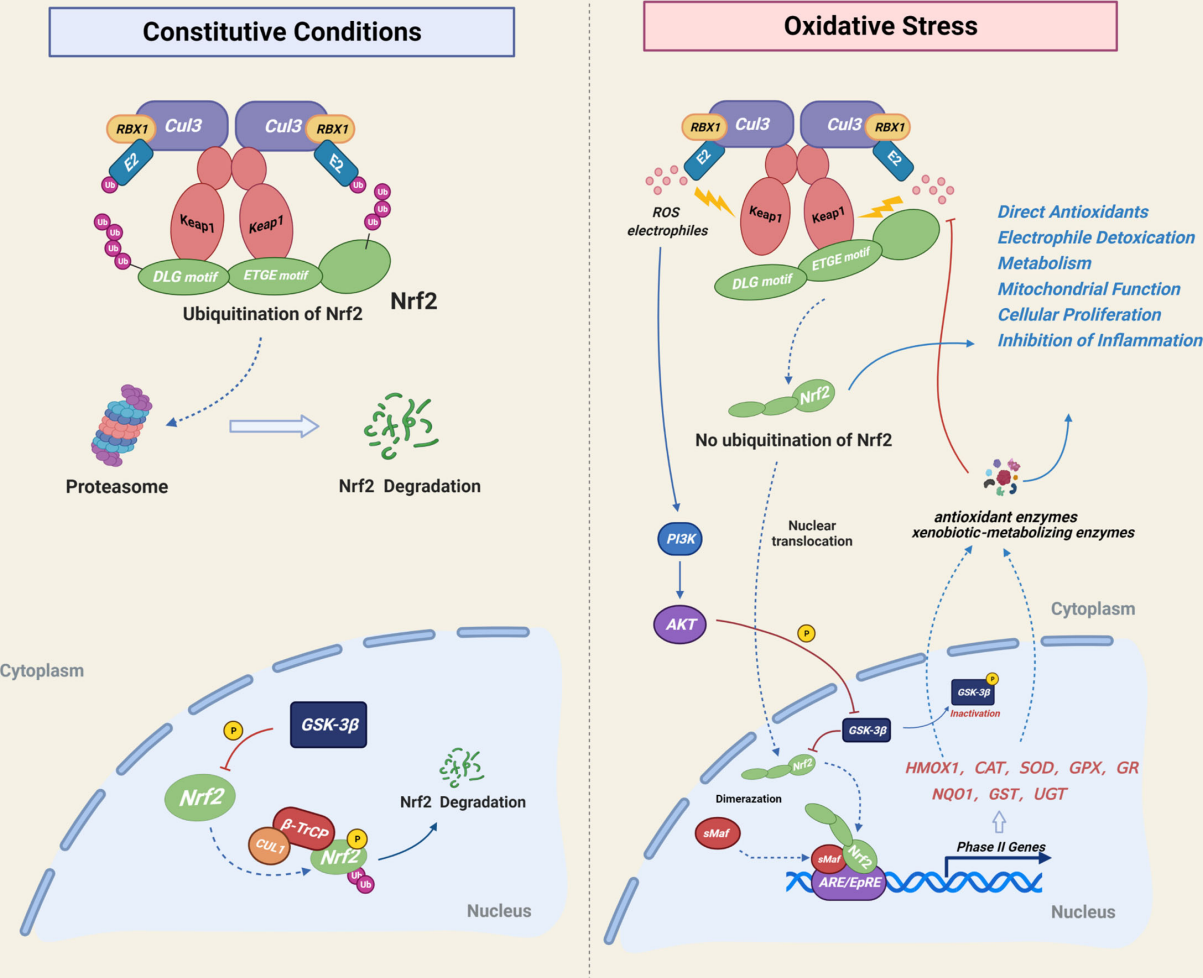
Mechanistic illustration of cellular NRF2 regulatory mode. Under basal unstressed conditions, NRF2 is degraded by Keap1-CUL3-mediated ubiquitination in the cytoplasm and by GSK-3β-CUL1-mediated ubiquitination in the nucleus. Under oxidative stress conditions, cysteine residues of Keap1 are modified by electrophiles or ROS, resulting in Keap1 deactivation and inhibition of the cytoplasmic ubiquitination of NRF2, which is subsequently translocated into the nucleus. The PI3K-Akt signaling pathway inhibits the nuclear degradation of NRF2 by regulating GSK-3β phosphorylation. NRF2 activation resulted in increased expression of phase II detoxifying enzymes and antioxidant enzymes, which act downstream of NRF2 in order to maintain cellular redox homeostasis.

As a basic leucine zipper (bZIP) transcription factor, Nuclear factor (erythroid-derived2)-like 2 (NRF2, NFE2L2) was isolated originally as a homologous of the hematopoietic transcription factor NF-E2 p45 in 1994 (17, 18). It was first identified as a trans-acting element of the antioxidant response element (ARE) or electrophile response element (EPRE), which acts as a cis-response element regulating cellular antioxidant enzymes (HO-1, CAT, SODs, GPX, GR, etc) and affects the expression of phase II enzymes, such as exogenous biotransformation detoxifying enzymes (NQO1, GST, UGT and SULTS) (19, 20). NRF2 is mainly degraded by ubiquitination in the cytoplasm and ARE-dependent genes are expressed under normal cellular conditions. NRF2 is considered the checkpoint in the regulatory response of oxidative stress in the body (21, 22). The cytoplasmic ubiquitination of NRF2 is mediated by Kelch like-ECH-associated protein 1 (KEAP1), a protein that is conjugated to NRF2 and facilitates the subsequent ubiquitination of NRF2. Subsequently, NRF2 is transported to the proteasome for degradation. Following exposure to electrophiles or reactive oxygen species(ROS), the cysteine residues of KEAP1 are modified, resulting in conformational changes of KEAP1 (19). The ubiquitin ligase E3 activity of the KEAP1-CUL3 complex is destroyed and NRF2 ubiquitination is disrupted. Undegraded NRF2 and newly synthesized NRF2 enter the nucleus and activate downstream gene transcription with the dimerization of sMaf (23). KEAP1 regulates NRF2 ubiquitination by activating or shutting down ubiquitin ligase E3 activity due to its highly reactive cysteine residues, making it a highly efficient and sensitive redox biosensor (22). There is a weak regulatory mechanism of NRF2 inside the nucleus. NRF2 is phosphorylated by glycogen synthase kinase 3-beta (GSK-3beta) and forms a β-TrCP-CUL1 E3 ubiquitin ligase complex, which is subjected to proteasomal degradation. The NRF2 pathway is activiated by the PI3K/AKT-mediated phosphorylation of GSK-3beta under oxidative stress conditions (20, 22, 24). (**Figure 1**). In addition to redox homeostasis, NRF2 plays critical roles in anti-inflammatory condition, DNA repair, mitochondrial function, iron, lipid and glucose metabolism, cell proliferation, cell cycle and immune response (19, 20, 25, 26). The following review will discuss the roles of NRF2 in various skeletal cells, as well as the effects of NRF2 inhibition and hyperactivation on animal bones.

## 2 NRF2 and Bone Cells

### 2.1 The Role of NRF2 in Osteoblasts

#### 2.1.1 NRF2 Inhibits Osteogenesis Differentiation of Osteoblast

NRF2 hyperactivation negatively regulated osteogenesis differentiation by inhibition of the RUNX2-dependent transcriptional activity in MC3T3-E1 cells. The levels of *Runx2* mRNA did not change significantly during the process, and there is no direct interaction between NRF2 and RUNX2, which may be explained by the inclusion of other proteins between them. In addition, NRF2 can directly bind to the ARE-like sequence near OSE2 in the osteocalcin promoter to reduce the transcriptional activity of osteocalcin as well (27). NRF2 hyperactivation in primary osteoblasts from *Keap1* knockout (*Keap1^−/−^*) mice leads to impaired osteogenic differentiation (28, 29). *Nrf2* knockdown or knockout significantly enhanced osteoblast mineralization (30). The primary osteoblasts from *Nrf2* knockout (*Nrf2^−/−^*) mice in two studies induces upregulation of the levels of the mRNA for *Runx2*, *Alpl* (alkaline phosphatase)*, Bglap* (osteocalcin)*, Sp7* (osterix) *or spp1* (osteopontin) (29, 30).

**Figure 2:**
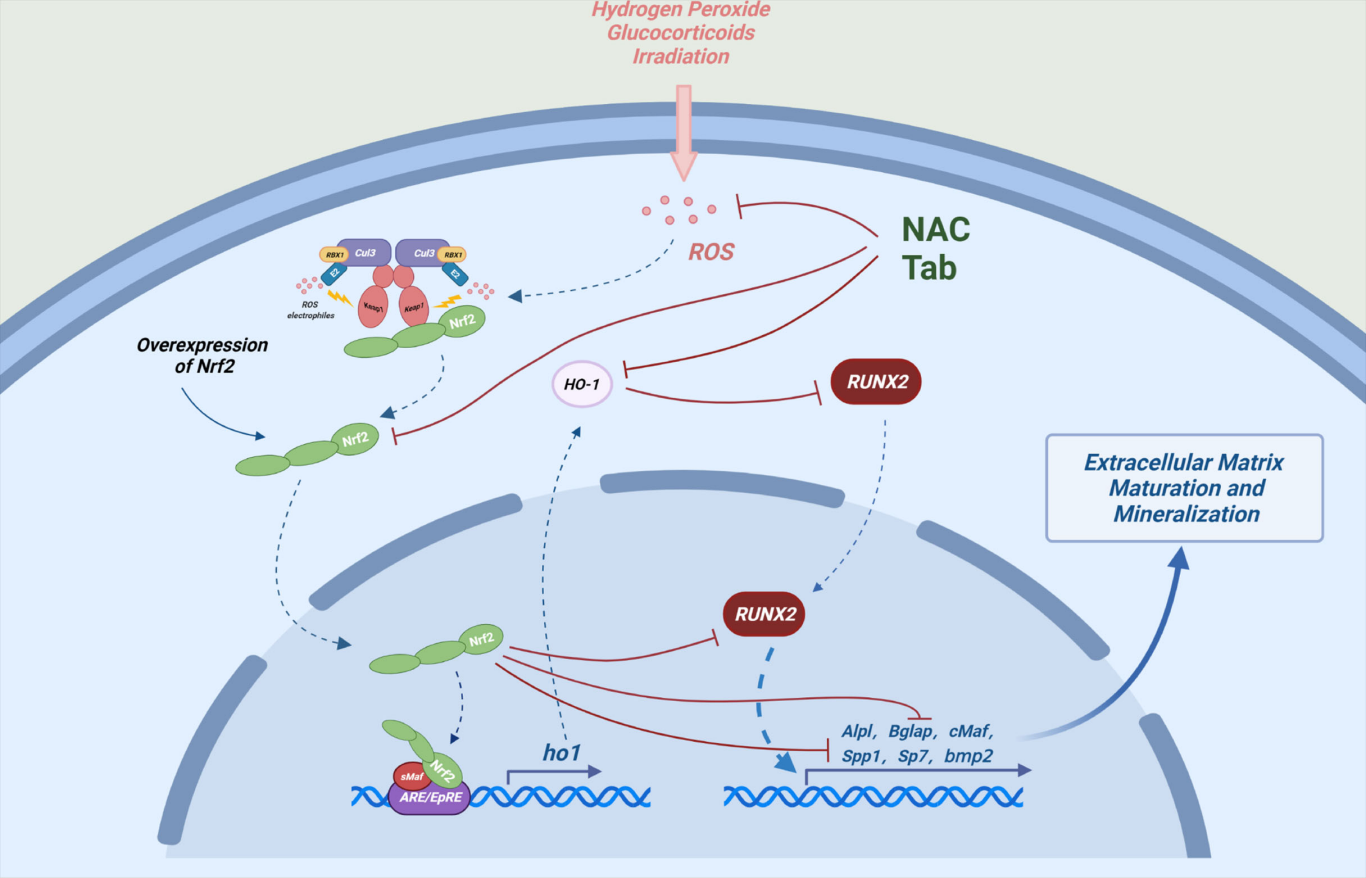
Possible mechanisms by which NRF2 inhibits osteoblast differentiation: Under oxidative stress, overexpression of NRF2 and its downstream factor (HO-1) can interact with Runx2, an important transcription factor in the process of osteogenic differentiation and can also directly bind to the ARE-like sequence near OSE2 in the osteocalcin promoter to cooperatively reduce the transcriptional activity of osteocalcin (Bglap) and ultimately inhibit the expression of a number of key genes regulating osteogenic differentiation and mineralization.

During oxidative stress conditions, upregulation of NRF2 may inhibit osteoblast differentiation. Low concentration of non-toxic H2O2 may inhibit differentiation and mineralization of MC3T3-E1 cells, which is accompanied by up-regulation of *Nrf2* gene expression (31). N-acetylcysteine (NAC), a precursor of glutathione (GSH), mitigated the inhibition of osteoblast differentiation and mineralization by H_2_O_2_ or radiation-induced oxidative stress, accompanied by down-regulation of the NRF2/HO-1 pathway, however, these studies did not conduct rescue experiments to prove that the effects of NAC depended on the inhibition of NRF2 (32, 33). Some studies have shown that NAC may reduce oxidative stress level through non-NRF2 activation pathway, thus alleviating the damage of osteoblast differentiation, meanwhile, the activation of the NRF2 itself and NRF2-driven stress genes, were inhibited by NAC (34, 35). In addition, the downstream antioxidant enzyme of NRF2 may also affect osteoblastic differentiation. Heme oxygenase-1(HO-1), which is the downstream target protein of NRF2, can inhibit the maturation and mineralization of osteoblasts (32, 33, 36). Although NRF2 inhibition alleviates the radiation-induced osteogenic differentiation and mineralization damage, ZnPP IX, an inhibitor of HO-1, significantly restored radiation-induced osteogenic damage without inhibiting radiation-mediated activation of NRF2 (33). Quercetin alleviates H_2_O_2_-induced damage to osteoblast differentiation by downregulating HO-1 without effecting on NRF2 expression (37). These data suggest that the relationship between osteoblastogenesis and NRF2/HO-1 or ROS is rather complex (**Figure 2**), and HO-1 may be a bigger culprit of oxidative stress-induced osteogenic injury.

NRF2 is closely related to autophagy in the process of osteogenic differentiation. During osteoblast differentiation in human osteoblasts (HFOB 1.19), the expression levels of type 2 cannabinoid receptor (CNR2) are increased, which activates autophagy and decreases the expression levels of p62, the latter can compete with NRF2 for binding to KEAP1 (38). CNR2 agonists promote osteogenesis and autophagy, while inhibiting NRF2 entry into the nucleus (39). However, some studies have shown that KEAP1 stabilizes NRF2 *via* the P62-dependent autophagy degradation pathway (40).

#### 2.1.2 NRF2 Promotes Osteogenesis Differentiation of Osteoblast

NRF2 has also been shown to promote the differentiation of osteoblasts. The increased ALP activity in osteoblast differentiation and matrix mineralization promotes the levels of free phosphate and stimulates mRNA levels of *Nrf2*, which may be explained by conferring protection for the cells survival in the harsh environment caused by mineralization (41). Moderate NRF2 activation (*Keap1* ^+^ *^/–^*) or hyperactivation of NRF2 (*Keap1^–/–^*) enhanced gene expression of *Alpl* and *Wnt5a* in primary osteoblasts (42). The expression levels of *Runx2* and *Col1a1* were decreased in *Nrf2* knockout primary osteoblasts (43). Another study showed that the number of osteoblasts colony-forming units and the mineralization of primary calvarial osteoblast from *Nrf2^−/−^* mice were significantly reduced compared with wild type (44). In addition, a study have indicated that the proliferation and differentiation of primary osteoblasts derived from *Nrf2^−/−^* mice are not significantly changed (45).

**Figure 3:**
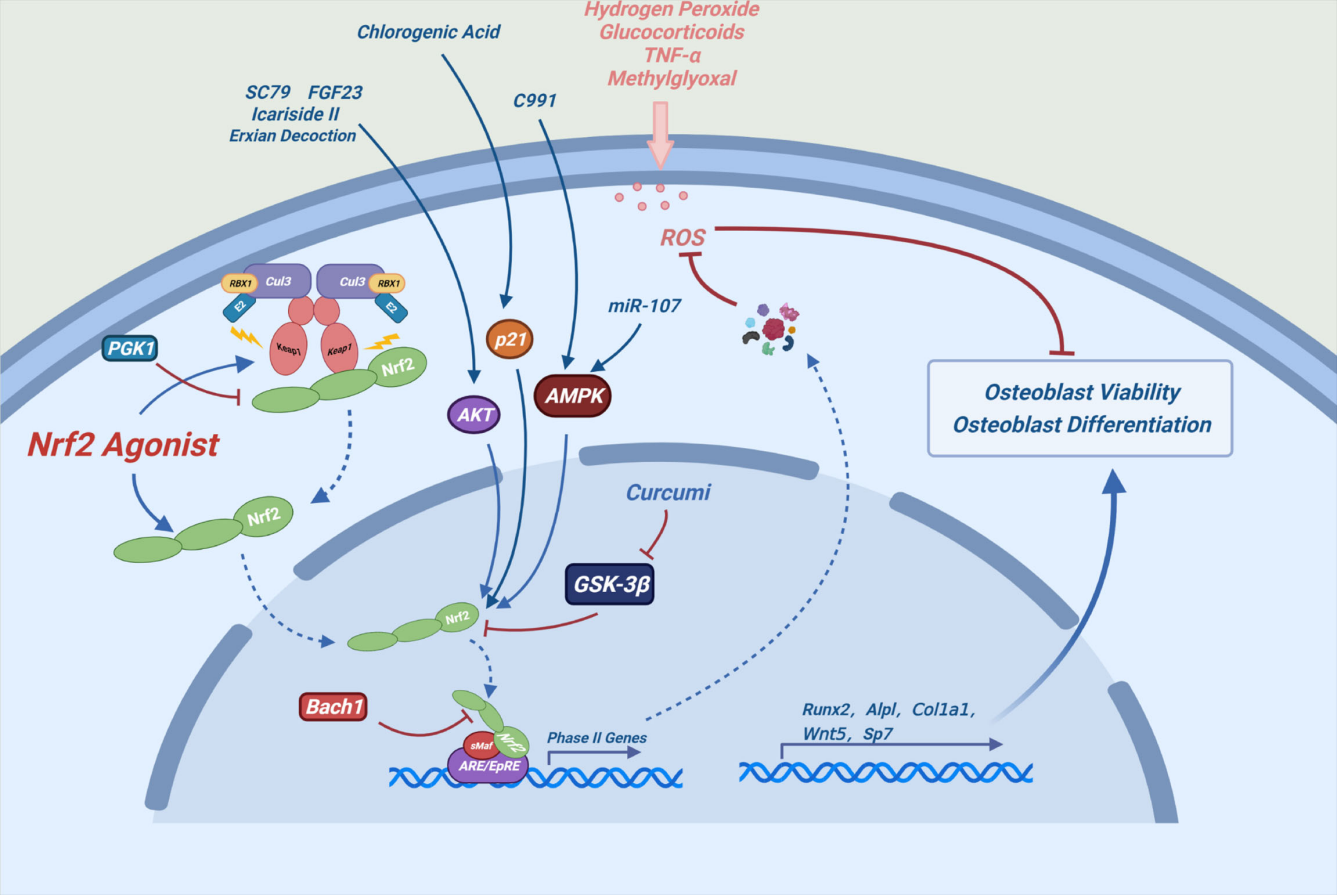
Possible mechanism of NRF2 promoting osteoblast differentiation: Under oxidative stress conditions, the expression levels of NRF2 and its downstream antioxidant enzymes can maintain redox homeostasis of cells and alleviate the damage of osteoblast differentiation and the toxic effects caused by oxidative stress in osteoblasts.

Certain drugs can alleviate oxidative stress-induced osteogenic differentiation damage by activating NRF2. Aucubin can alleviate the H_2_O_2_-induced osteogenic differentiation inhibition *via* the NRF2 signaling pathway (46). Bach1 is a competitive inhibitor of NRF2, which impairs cellular activity and function of MC3T3-E1 cells. BACH1 silencing can inhibit the production of reactive oxygen species (ROS) and promote osteoblast differentiation by enhancing the NRF2/ARE signaling pathway (47). Aucubin, gastrodin, Z-guggulsterone, proanthocyanidins and can prevent the osteogenic differentiation damage induced by glucocorticoids by activating the NRF2 pathway (46, 48–50) (**Figure 3**).

#### 2.1.3 NRF2 Protects Osteoblasts From Oxidative Stress-Induced Cytotoxicity

Many chemicals alleviate oxidative stress-induced cytotoxicity by activating the NRF2 signaling pathway in osteoblasts. For example, luteolin, melatonin and glabridin can ameliorate the methylglyoxal or high glucose-induced cytotoxicity in osteoblasts by activating the NRF2/ARE signaling pathway (51–53). Sulforaphane, indole-3-carbinol, plumbagin and alpinumisoflavone can ameliorate glucocorticoid-induced cytotoxicity of osteoblasts by activating the NRF2 signaling pathway (54–57). Dex significantly reduced the half-life of the cell cycle-related protein P21WAF1/CIP1 by inhibiting the AKT signaling pathway in osteoblasts. Depletion of P21 inhibits NRF2/HO-1 signaling pathway, increasing ROS production and osteoblast damage (58). Chlorogenic acid plays a osteogenic protective role by relieving the inhibition of Dex on P21 and by activating the NRF2 signaling pathway (59). The AKT activator SC79, Icariside II and FGF23 can alleviate the Dex-induced cytotoxicity by activating the AKT-NRF2 signaling pathway (60–62). miR-107 and the AMPK activator compound 991 can activate AMPK-NRF2 signaling pathway to resist Dex-induced oxidative stress damage (63, 64). These results indicate that the AKT-NRF2 and AMPK-NRF2 signaling pathways play an important role in these processes. In addition, inhibition or depletion of phosphoglycate kinase 1 (PGK1) leads to accumulation of methylglyoxal, which modifies KEAP1 to activate the NRF2 pathway and protect against Dex-induced damage in osteoblasts (65). δ-tocotrienol, stilbene glycoside, mangiferin and fermented oyster extracts can relieve H_2_O_2_-induced damage by activating the NRF2 pathway in osteoblasts (66–69). Exian decoction can reduce the damage of TNF-α to osteoblasts by activating the AKT-NRF2 pathway (70). In addition, A phenolic acid phenethyl urea derivative can ameliorate the radiation damage by activating the NRF2 pathway (71).

### 2.2 The Role of NRF2 in Stem Cells

#### 2.2.1 The Role of NRF2 in Osteogenic Differentiation of Stem Cells

The role of NRF2 in osteogenic differentiation of stem cells remains controversial. The colony formation ability of bone marrow mesenchymal stem cells (BMSCs) derived from *Nrf2^−/−^* mice was significantly reduced, resulting in a significant reduction in the number of osteoblasts in 3-week-old *Nrf2^−/−^* mice (45).Nuclear abundance of NRF2 was decreased during adipogenesis of mouse bone marrow stromal cells (ST2 cells), which indirectly demonstrated the promoting effect of NRF2 on osteogenic differentiation of stem cells (72). In addition, hypoxic environment is conducive to the maintenance of stemness and the osteogenic ability of BMSCs. *Nrf2* knockout resulted in impaired stemness and osteogenic ability of BMSCs under hypoxia. It has been suggested that NRF2 activation may be a potential method to maintain the stemness of BMSCs under hypoxia conditions (73).

NRF2 may also have a negative effect on osteogenic differentiation of stem cells. Pretreatment of the human periodontal ligament fibroblasts (hPLFs) with the Wnt agonist lithium chloride (LiCl) or Wnt1 can alleviate H_2_O_2_-induced osteogenic differentiation and mineralization damages with almost complete inhibition of NRF2, and siRNA-mediated NRF2 inhibition restored these injuries (74).

A certain concentration of ROS can promote bone formation and the role of NRF2 in this process is contradictory. Osteogenic differentiation of adipose derived stem cells (ADSCs) was accompanied by NRF2 downregulation and autophagy activation, ROS produced by H_2_O_2_ promoted osteogenic differentiation of ADSCs, and further up-regulated of autophagy and NRF2 levels. The effects were enhanced by inhibition of NRF2 and reversed by inhibition of autophagy (75). In contrast, mild oxidative stress induced by low concentration of glucose oxidase (GO) promotes the expression levels of NRF2 and HO-1 in embryonic stem cells (ES), while enhancing the osteogenic differentiation and mineralization, which is reversed by inhibition of NRF2 (76). Deferroamine (DFO) promotes osteogenic differentiation of human periodontal ligament cells (hPDLCs) by increasing ROS concentration, accompanied by the activation of NRF2, and the effects were attenuated by NRF2 inhibition (77).

Many drugs or chemicals can promote osteogenic differentiation of stem cells by the activation of the NRF2 signaling pathway. Dendrobium officinale polysaccharides (DOPs) significantly promoted osteoblast differentiation and inhibited adipogenic differentiation of BMSCs by activating NRF2 pathway (78). The novel neohesperidin dihydrochalcone analogue inhibits adipogenic differentiation of human adipose-derived stem cells *via* the NRF2 pathway as well (79). Panax ginseng fruit protects against porphyromonas gingivalis lipopolysaccharide (PG-LPS)-induced osteoblastic differentiation injury in hPDLCs by activating the NRF2/HO-1 signaling pathway (80). Metformin alleviates H_2_O_2_-induced osteogenic differentiation damage of periodontal ligament stem cells (PDLSCs) by activating NRF2 signaling pathway (81). The inhibition of nuclear import of NRF2 by Ochratoxin A (OTA) resulted in the loss of the maintenance of self-renewal capacity and differentiation potential in the osteogenic lineage of human mesenchymal stem cells (hMSCs), while enhanced nuclear import of NRF2 by tert-butylhydroquinone (t-BHQ) had opposite effects (82).

#### 2.2.2 NRF2 Protects Stem Cells From Oxidative Stress-Induced Cytotoxicity

The protective effect of NRF2 activation on stem cells has been confirmed by several studies. The interference of KEAP1 expression in adipo-derived mesenchymal stem cells (AD-MSCs) by using the CRISPR/Cas9 method, or directly over expressed NRF2, which can significantly enhance the anti-oxidative ability of stem cells (83, 84). Estradiol (E2) activates NRF2/SIRT/Mn SOD3 pathway in human umbilical cord blood mesenchymal stem cells (hUCB-MSCs), which protects cells from high glucose-induced mtROS production and autophagic cell death (85). On the other hand, Triclosan (TCS) induces cytotoxicity *via* disrupting the nuclear translocation of SKN-1/NRF2 in hMSCs (86). Polydatin, Coenzyme Q10 and Fufang Lurong Jiangu capsule can effectively prevent the activity decline of BMSCs induced by H_2_O_2_ *via* activating the NRF2 pathway (87–89). In addition, BMSCs pretreated with a low dose of non-toxic concentrations of H_2_O_2_ enhanced the ability of BMSCs to resist oxidative stress injury induced by high concentrations of H_2_O_2_ by activating the NRF2 signaling pathway, which demonstrated the central role of NRF2 in the electrophile counterattack response (22, 90).

### 2.3 The Role of NRF2 in Osteoclasts

A number of studies performed on primary cells or cell lines have fully demonstrated that NRF2 hyperactivation can inhibit osteoclast differentiation, while NRF2 inhibition exhibits the opposite effects (29, 30, 44, 45, 91–95). RANKL binds to RANK to induce various intracellular signal transduction cascades *via* TRAF-6, in which induction of ROS generation are crucial for osteoclast formation (96–98). The regulation of NRF2 on oxidative stress may be the main mechanism of involved in osteoclast differentiation (44). Activation of the NRF2/ARE signaling pathway can regulate osteoclast differentiation by controlling intracellular ROS signaling.

**Figure 4:**
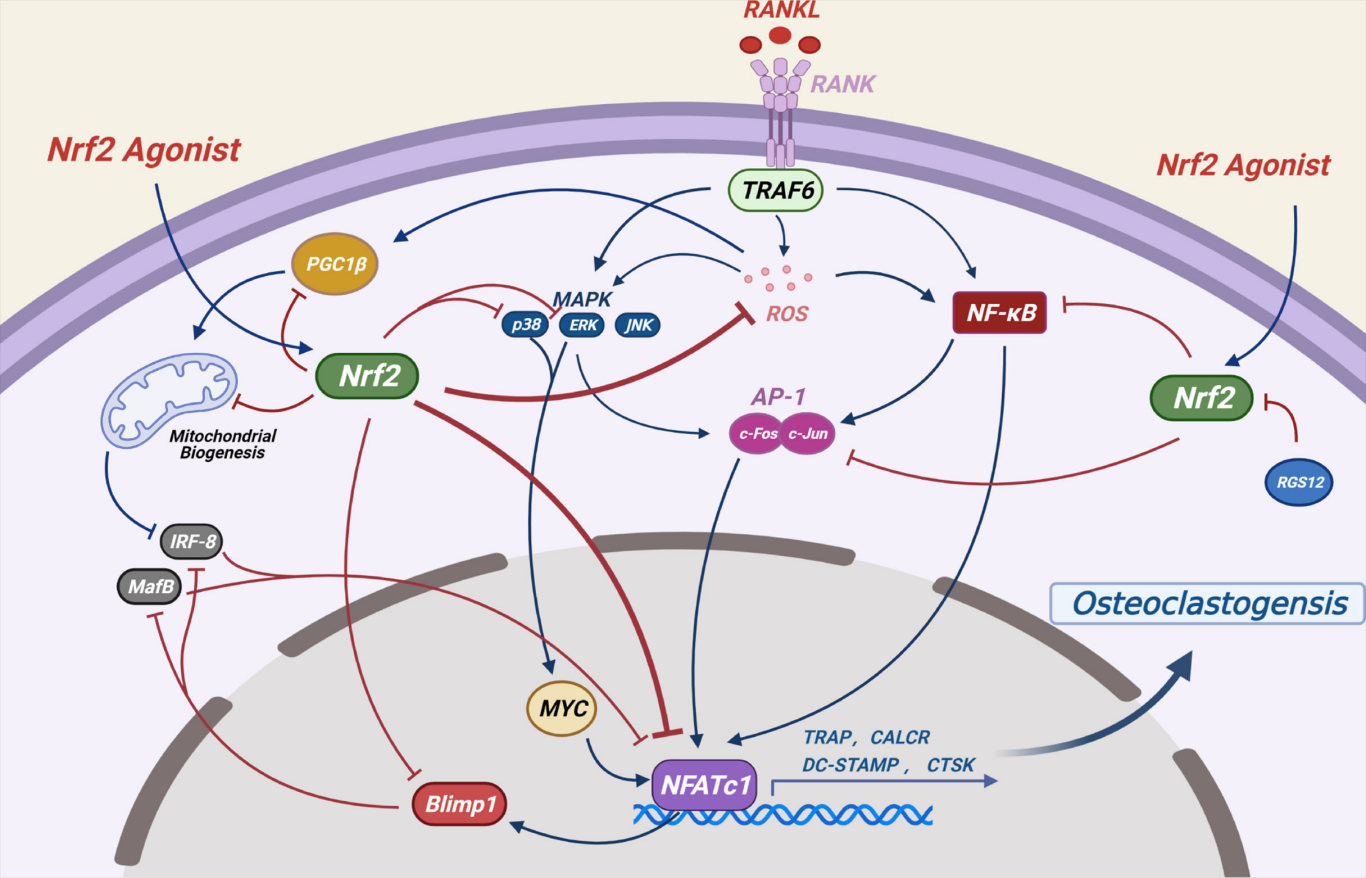
Potential mechanisms by which NRF2 inhibits osteoclast differentiation: RANKL-induced ROS production and several other signaling pathways are crucial to osteoclast formation. By regulating the production of ROS and the expression levels of NF-κB, MAPKs, AP-1 as well as mitochondrial generation and other related signaling pathways, the NRF2/ARE signaling pathway ultimately inhibits the activity of NFATC1, a key transcription factor for osteoclast differentiation, thereby inhibiting osteoclast differentiation. (Dotted blue arrows indicate molecular displacement; the solid blue arrow indicates the subsequent effect; the red “T” line indicates inhibition).

NRF2 inhibits ROS-mediated MAPK activation (ERK, P38 and JNK) (29), which are key signaling molecules for osteoclast formation (6) (**Figure 4**). As a key regulator of osteoclast generation, MYC can directly regulate the activity of NFATc1 (99). It has been reported that NRF2 inhibits the levels of MYC by inhibiting the activation of ERK and P38 signals, ultimately impeding osteoclast generation (93).

NF-κB is the first eukaryotic transcription factor to be demonstrated in response to ROS and oxidative stress (100), its role in the regulation of osteoclast differentiation by NRF2 has been controversial. It has been shown that *Nrf2*-knockdown or knockout significantly upregulated NF-κB transcriptional activity and promoted osteoclast differentiation in RANKL-induced bone marrow derived macrophages (BMMs) (30), However, in another study, *Nrf2*-knockout promoted RANKL-induced osteoclast differentiation in BMMs without NF-κB activation (92). In addition, hyperactivation of NRF2 in *Keap1*-knockout spleen macrophages induced by RANKL significantly reduces nuclear translocation of NF-κB (29).

The dimer AP-1, which is mainly composed of Fos and Jun proteins, plays an important role in osteoclast formation (101). In the initial stage of RANK signal transduction, activation of NF-κB and MAPKs leads to activation of c-FOS, which further promotes osteoclast formation (102, 103). Knockdown or knockout of *Nrf2* resulted in upregulation of c-FOS levels of RANKL-induced BMMs (30, 92), and NRF2 hyperactivation resulted in decreased phosphorylation of c-FOS by RANKL-induced splenic macrophages into the nucleus (29). The inhibitory effect of NRF2 on c-Fos may be due to its negative regulation of NF-κB and its direct binding with c-Fos promoter (30).

NFATC1 is the main switch regulating the final differentiation of osteoclasts. It regulates the expression of osteoclast specific genes such as tartrate-resistant acid phosphatase (TRAP), calcitonin receptor (CALCR), cathepsin K (CTSK), dendritic cell-specific transmembrane protein (DC-STAMP) and αVβ3 integrin during the final stage of osteoclast differentiation (104). RANKL-induced NF-κB, AP-1 and other proteins are recruited to the promoter region of the NFATc1 gene to jointly induce the expression levels of NFATc1 (104, 105). NRF2 may eventually regulate the expression of NFATc1 by affecting the aforementioned classical signaling pathways and plays a role in osteoclast regulation. Since NFATc1 is an important transcriptional regulator acting downstream of c-FOS (105), *Nrf2*-knockdown or knockout leads to the upregulation of NFATc1 levels in RANKL-induced BMMs (30, 92). Moreover, *Keap1* -knockout in splenic macrophages results to the complete elimination of RANKL-induced NFATc1 expression (29).

The transcription factors MafB and interferon regulatory factor 8 (IRF-8) inhibit the activation of NFATc1 during osteoclast differentiation. NFATc1 induces the activation of B lymphocyte-induced maturation protein 1 (BLIMP1, encoded by *Prdm1*), a transcriptional repressor of these two antiosteoclast genes (106–108). Peroxisome proliferator–activated receptor-γ coactivator 1β (PGC-1β) induces the inhibition of IRF-8 and promotes the differentiation of osteoclasts by inducing mitochondrial biogenesis (109, 110). NRF2 hyperactivation in spleen macrophages from *Keap1^–/–^* mice completely eliminated the expression of *Prdm1* and upregulated the expression of *Mafb*. The wild-type (WT) and *Nrf2^–/–^* cells demonstrated the opposite effects. NRF2 hyperactivation inhibited oxidative stress and decreased the expression levels of *Ppargc1b* and of specific mitochondrial genes, leading to significant upregulation of *Irf-8* expression, resulting in blocked activation of NFATc1 and osteoclast differentiation (29).

Previous studies on the regulatory factors of NRF2 have also produced similar conclusions. The inhibition of the NRF2 activator dipeptidyl peptidase 3 (DPP3) resulted in increased oxidative stress levels and enhanced osteoclast activity (111). The upstream regulator of NRF2, the regulator of G protein signaling 12 (RGS12), promoted the formation of osteoclasts by inhibiting NRF2 and increasing ROS levels (112). RANKL induces BTB and CNC homology 1 (BACH1), a competitive inhibitor of NRF2, into the nucleus and weakens the expression levels of NRF2-mediated antioxidant enzymes to promote osteoclast generation, which can be reversed by BACH1 inhibitor treatment (113, 114). In addition, inhibition of NRF2 in RAW 264.7 cells or bone marrow macrophages (BMMs) promoted arsenic-induced osteoclast differentiation (115).

It is interesting that no significant differences in the number of osteoclasts or bone resorption activity have been noted in *Nrf2^–/–^* mice (29, 30, 45). Previous studies have shown that osteoclast formation of WT or *Nrf2^–/–^* BMMs is significantly reduced following co-culture with *Nrf2^–/–^* osteoblasts (30). Osteoclast differentiation of WT macrophages was not inhibited following co-culture with *Keap1^–/–^* osteoblasts (29). Some studies have suggested that NRF2 inhibits the levels of OPG in osteoblasts by binding to the OPG promoter, therefore, *Nrf2^–/–^* osteoblasts showed increasing OPG levels, affecting the RANKL/OPG ratio and reducing osteoclast formation *in vivo* (30). In addition, co-culture of *Nrf2^–/–^* osteoblasts with WT/*Keap1^–/–^*/*Nrf2^–/–^* macrophages resulted in lower osteoclast formation compared to what was observed in co-culture of WT macrophages with WT osteoblasts, these results confirmed the important role of NRF2 in indirectly regulating osteoclast differentiation by influencing osteoblasts (29). However, Another study show that LPS induced the expression of IL-6 in MC3T3-E1 cells, while the activation of NRF2 in osteoblasts can inhibit the expression of IL-6 and in turn indirectly inhibit the generation of osteoclasts (116).

**Figure 5:**
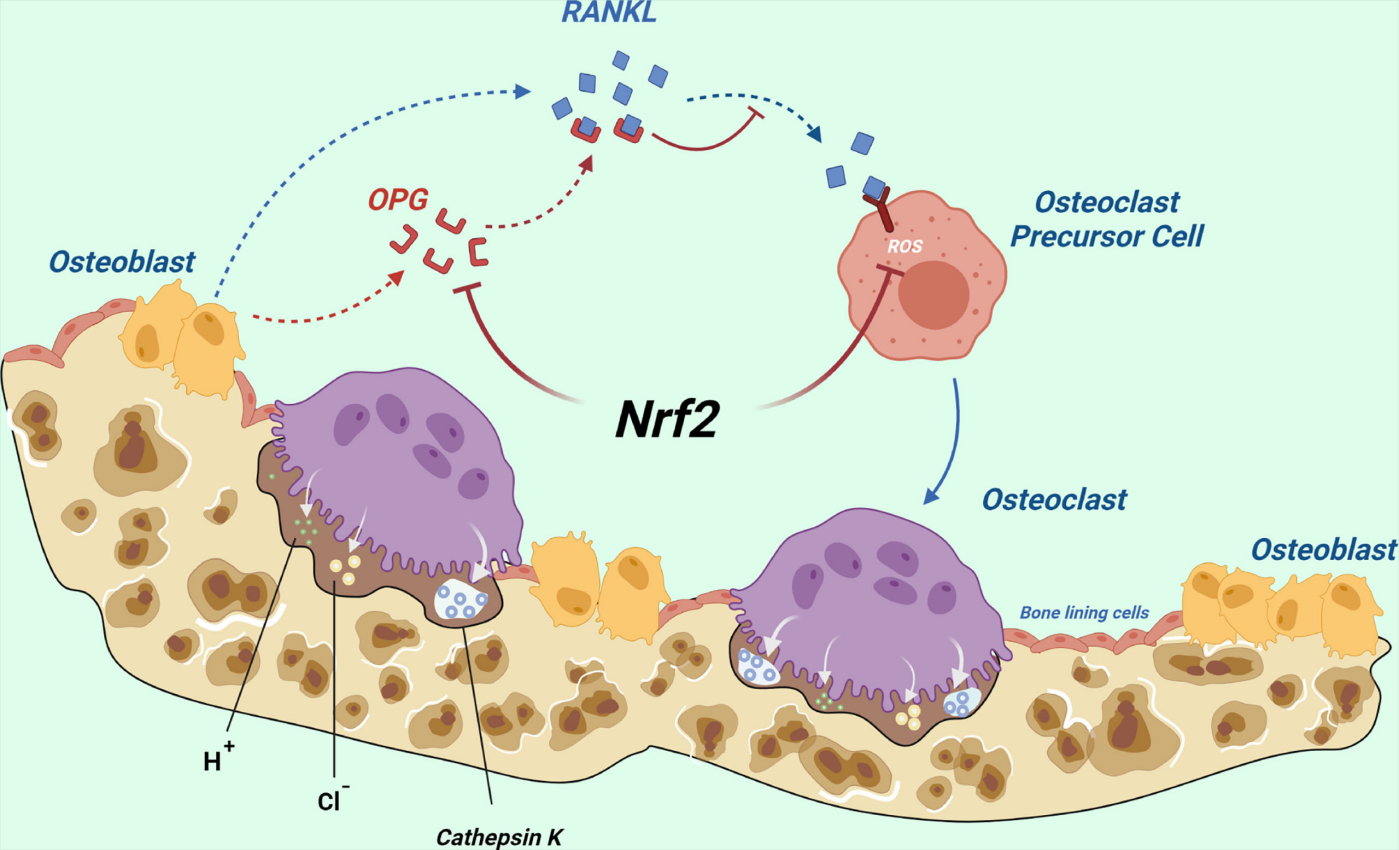
Total effects of NRF2 on osteoclast differentiation *in vivo*: NRF2 can directly inhibit osteoclast differentiation by decreasing ROS production, whereas it can also indirectly promote osteoclast formation by inhibiting the secretion of OPG by osteoblasts. The latter may play a major role *in vivo*. (Dotted blue arrows indicate molecular displacement; the solid blue arrow indicates the subsequent effect; the red “T” line indicates inhibition).

NRF2 can inhibit osteoclast differentiation directly by decreasing ROS production, whereas it can also indirectly promote osteoclast formation by inhibiting the secretion of OPG in osteoblasts (**Figure 5**). It may play a major role *in vivo*.

Studies on the mechanism of drugs inhibiting bone resorption suggest that NRF2 activation may be the key to inhibiting bone resorption. The NRF2 inducers, including Bardoxolone methyl (CDDO-ME), Sulforaphane (SFN) and tert-butylhydroquinone (tBHQ) can activate NRF2 and its downstream antioxidant genes to downregulate RANKL-induced ROS levels and inhibit osteoclast formation (117). The NRF2 activator RTA-408 attenuates osteoclast generation by inhibiting STING-dependent NF-κB signal transduction (118). miR-1225 can inhibit osteoclast formation by targeting *Keap1* mRNA degradation (119).

### 2.4 The Effects of NRF2 on Osteocytes

It indicated that during the fusion of IDG-SW3 cells into osteocytes and the differentiation of primary osteoblasts into osteocytes, significant enhancement of mitochondrial biogenesis leads to an increase in reactive oxygen species (ROS) levels accompanied by an increase in NRF2 activity. The expression levels of the osteogenic genes *Runx2*, *Sp7* and *Col1a1* were only slightly decreased in primary osteocytes from osteoblast-specific *Nrf2^–/–^* mice, while the expression levels of the osteogenic genes, such as *Dmp1*, *Mepe* and *Sost* were significantly decreased. Following hyperactivation of NRF2, the gene levels of the suppressed bone cells were significantly upregulated (43). The NRF2 activator dimethyl fumarate (DMF) exhibited no significant effect on the expression of *Runx2* and *Col1a1* in WT osteocytes. However, the expression levels of the osteocyte-specific genes *Dmp1*, *Mepe* and *Sost* were obviously increased. Additional bioinformatic and genomic analyses indicated that NRF2 activated the expression of these genes by directly binding to the regulatory regions of *Dmp1*, *Mepe* and *Sost*. Due to dimethyl fumarate (DMF) could not alleviate the osteocytic genes inhibition in *Nrf2^–/–^* osteocytes, demonstrating that NRF2 leads to transcriptional activation of these genes (43). Meanwhile, alpinumisoflavone can alleviate glucocorticoid-induced osteocytes injury by activating the NRF2 pathway (57).

### 2.5 Effects of NRF2 on Chondrocytes

Hyperactivation of NRF2 inhibited chondrogenic differentiation of mouse pre-chondrogenic cell line ATDC5 cells. The levels of type II collagen, type X collagen and osteopontin, which are markers of chondrogenic differentiation, were significantly decreased, indicating that NRF2 exhibited an inhibitory effect on chondrogenic formation (120). NRF2 plays an important role in cartilage protection in the progression of osteoarthritis. *Nrf2^–/–^* mice showed more severe cartilage damage in OA model (121). The levels of NRF2 and its downstream proteins in cartilage of the same patient was significantly higher than that in normal cartilage and the expression in the affected area was significantly higher than that in the smooth area. NRF2 may act as a stress response protein of OA chondrocytes by significantly inhibiting the activation of IL-1β-induced exogenous and endogenous apoptotic pathways (122). Some drugs relieve osteoarthritis by activating the NRF2 signaling pathway to reduce inflammation and induce extracellular matrix (ECM) degradation of chondrocytes *in vivo* and *in vitro* models of OA (123).

## 3 Role of NRF2 in Animal Bone

### 3.1 NRF2 Inhibition/Activation and Bone in Animal

#### 3.1.1 NRF2 Knockdown/Knockout and Bone in Animal

Bone acquisition or loss, the number and activity of osteoblasts and osteoclasts in *Nrf2^–/–^* animal models are controversial. Most studies have shown that *Nrf2* knockout impairs animals bone. NRF2 is critical for animal bone development after birth. In female *Nrf2^–/–^* mice, increased oxidative stress significantly inhibits the colony formation ability of BMSCs, resulting in impaired osteoblast formation, manifested as significantly impaired bone acquisition at 3 weeks of age. The bone mass gradually returning at 12 weeks. The contribution of other antioxidant pathways *in vivo* that are not dependent on NRF2 signaling, such as FOXO, may compensate for the loss of NRF2 (45). As previously mentioned, several studies have shown that NRF2 regulation of RANKL/OPG does not cause significant difference in mouse osteoclast numbers and activity (29, 30, 45). However, significant upregulation of osteoclast numbers and activity in *Nrf2^–/–^* mice has also been observed in several studies (95, 124). Female *Nrf2^–/–^* mice aged 6 to 9 months exhibited higher osteoblast numbers and activity than WT mice, however, the increased RANKL/OPG ratio resulting in the more osteoclast numbers, the stronger activity of osteoclasts and the bone conversion rate, which ultimately led to bone loss (95). Compared with age-matched WT mice, bone formation and mineralization in male *Nrf2^–/–^* mice at 17 weeks of age were significantly decreased, and the numbers and surface of osteoclasts were significantly increased, leading to severe bone loss (124).

NRF2 inhibition has also been observed to benefit bone acquisition in animals. *Nrf2^–/–^* mice at 9 weeks of age exhibited higher bone mass, higher mineral attachment rate, higher level of serum osteocalcin and the number of osteoblasts than WT mice (30).

In addition, a study showed that the number of osteoblasts decreased and the number of osteoclasts increased in sex-matched *Nrf2^–/–^* mice at 3 months of age, but bone volume did not change significantly (44). Another study also found that bone parameters of *Nrf2^–/–^* female mice were similar to those of WT mice (94).

NRF2 may be indispensable in the protection of bones against environmental stress damage. Irradiation (IR) induced more severe bone loss in *Nrf2^–/–^* mice compared to WT mice. Further studies have shown that NRF2 plays a critical role in osteoblast differentiation and stromal formation regardless of IR presence (44). NRF2 inhibition reduce load-driven bone formation. The parameters, such as femur bone mineral density, bone formation rate and ultimate force as well as relative mineralizing surface and relative bone formation rate were significantly reduced in *Nrf2^–/–^* mice (124). NRF2 plays an important role in bone regeneration. NRF2 is activated in the process of fracture healing. *Nrf2^–/–^* mice suffered from more brittle bone cortex and significantly reduced anti-fracture force and anti-bending stress. *Nrf2*-knockout induced aggravation of oxidative damage of cartilaginous callus in femoral shaft fracture model mice, resulting in delayed bone healing and remodeling. It is worth mentioning that the expression of osteocalcin mRNA in the damaged tissues of *Nrf2^–/–^* mice was significantly lower than that of the WT mice, indicating that NRF2 binding to the osteocalcin promoter caused upregulation of osteocalcin expression *in vivo* (125), which is contradicted in previous study (27).

In addition, a study explored the differences in the effects of *Nrf2*-knockout on bone in male and female, old and young mice. The regulation of NRF2 on bone mass accumulation depends on sex and it is necessary in females, while dispensable or even deleterious in males. The total, femoral and spinal bone mineral density (BMD) of *Nrf2^–/–^* female mice was lower than that of female WT mice for at least a period of time, however, NRF2 deficiency inhibited bone loss in old male mice, suggesting that it negatively affected bone maintenance in male bones. In both young and old female mice, complete endogenous antioxidant responses (phase II detoxifying and antioxidant enzyme expression) were undertaken by NRF2-dependent mechanisms, whereas in older male mice, It was entirely undertaken by NRF2-independent mechanisms. And the NRF2-dependent phase II detoxifying enzyme expression and NRF2-independent oxidase expression coexist in male young mice. It means that the maintenance of the optimal antioxidant response in female bones depends on NRF2, whereas the aging skeleton of males does not require NRF2 for protection against oxidative stress (126).

Activation of NRF2 is critical for osteocytogenesis, through direct activation of certain osteocyte-specific genes (*Dmp1*, *Mepe*, and *Sost*) and osteoblastic gene (*Runx2* or *Os*x). Both sexes of osteocytic cell lineage-specific *Nrf2^–/–^* mice (*Dmp1/Nrf2*-KO) showed severe osteopenia. However, Osteoblast-specific *Nrf2^–/–^* mice (*Col1a1/Nrf2*-KO) indicated subtle osteopenia only in males, whereas no significant changes were noted in female bones. *Col1a1/Nrf2*-KO males and females showed the reduced expression of osteocyte-specific genes (*Dmp1*, *Mepe*, and *Sost*), but no significant changes in expression of osteoblastic genes (*Runx2* or *Os*x). The differences in osteogenesis gene expression may be due to the deletion period of *Nrf2* in osteocytic cell lineage is later than that in osteoblasts (Late embryo and neonatal period), the *Col1a1/Nrf2*-KO mice may have some compensatory mechanisms to restore the bone phenotypes. The number of osteoclasts was significantly increased in males but not females in both *Dmp1/Nrf2*-KO and *Col1a1/Nrf2*-KO mice. The sex difference complicated the effect of *Nrf2*-knockout in osteogenic lineages on paracrine secretion of osteoclasts by regulating RANKL/OPG ratio (43).

#### 3.1.2 NRF2 Hyperactivation/Moderate Activation and Animal Bone

NRF2 hyperactivation neonatal mice constructed from homozygous *Keap1* mutation died within three weeks due to severe hyperkeratosis in the esophagus and forestomach (127). One study observed that male neonatal *Keap1^–/–^* mice showed slight talus and calcaneus deformation and shortened growth plate hypertrophy, and the number of osteoclasts was decreased (29). Viable *Keap1^–/–^* mice were generated by the deletion of *Nrf2* in esophageal (NEKO mice), the data indicated that NEKO mice at the age of 8-10 weeks exhibited small body size and low bone density. As the differentiation of both osteoclasts and osteoblasts was weakened, it was suggested that the impaired differentiation of osteoblasts but not osteoclasts was the cause of bone hypoplasia in NEKO mice (28).

Moderate activation of NRF2 by heterozygous *Keap1* mutation(*Keap1^+/−^*) produced another viable *Nrf2*-overexpressed mice model. The bone phenotype of NRF2 moderate activation indicated sexual dimorphism. Bone formation was significantly increased and bone resorption was significantly reduced in male *Keap1^+/−^* mice compared to the littermate controls. And no significant effects were noted in females. In addition, The male *Keap1^+/−^* and *Keap1^−/−^* neonatal mice showed more bone mineralization in their limbs than WT mice, suggesting that hyperactivation and moderate activation of NRF2 could both accelerate bone mineralization (42). However, osteogenic differentiation was impaired in NEKO mice (28). In the future, osteoblast-specific *Keap1^−/−^* mice may aid to further elucidate differences in the role of KEAP1/NRF2 in bone formation (42). Briefly, more studies show that hyperactivation of NRF2 may cause damage to bone formation, while moderate activation of NRF2 promotes increased bone mass.

### 3.2 NRF2 and Ovariectomized Animal Models

Ovariectomy leads to increased oxidative stress levels due to reduction in estrogen levels. *Nrf2* mRNA levels was significantly up-regulated in osteoblasts of femur cancellous bone of ovariectomized mice (27). However, More studies have observed NRF2 was inhibited in the bone tissues of ovariectomized animal models and lutein, theaflavin-3, 3’-digallate, dimethyl fumarate, sodium butyrate and irisin could alleviate bone loss in ovariectomized animals by activating the NRF2 signaling pathway (43, 128–130). NRF2 inhibition further aggravates bone loss due to ovariectomies (94, 95). NRF2 inhibition accelerated the differentiation of osteoclasts in ovoectomized animals *in vitro*, whereas in *Nrf2^–/–^* female mice, which were ovariectomized, the oxidative stress levels were not exacerbated, suggesting that ROS-mediated osteoclast response was caused by NRF2 deficiency (95). A significant decrease in NRF2 levels was noted in the femur of ovariectomized mice and in osteoporosis patients possibly due to hypermethylation of the *Nrf2/NRF2* promoter mediated by abnormally elevated DNA methyltransferase (DNMT) 1/3a/3b. Appropriate physical exercise (like running) could alleviate this abnormality and prevents ovariection-induced bone loss, while *Nrf2-* knockout significantly attenuates exercise-induced bone protection (94).

### 3.3 Sex Dimorphism of NRF2 in Bone

Most studies show that NRF2 inhibition results in impaired bone mass in female mice (45, 95, 126), Osteocytic cell lineage-specific *Nrf2^–/–^* is harmful to bone mass in female mice (43). However, it showed that no significant changes were noted in the bone volume or bone mass of female *Nrf2^–/–^* mice compared with that of WT mice (44, 94). Osteoblast-specific *Nrf2*-knockout did not significantly affect the bone mass of female mice as well (43). While moderate NRF2 activation had no significant effect on female mice (42).

The effect of NRF2 inhibition on bone of male mice is highly controversial. A study reported NRF2 inhibition was beneficial to bone mass in male mice (126). but another study exhibited severe bone loss in male *Nrf2^–/–^* mice compared with WT mice (124). The osteocytic cell lineage-specific *Nrf2*-knockout caused severe bone loss in male mice, and the osteoblast-specific *Nrf2*-knockout only caused slight bone damage in male mice (43). In addition, *Nrf2*-knockout may have no significant effects on male skeleton (44). On the other hand, certain studies have reported that NRF2 hyperactivation impairs the bone phenotype of male mice (28, 29). And moderate activation of NRF2 significantly accelerated bone formation and mineralization and increased bone mass in male mice (42).

NRF2 appears to be indispensable or harmless for bone acquisition in female mice and its role in male bone is contradictory. The interaction of NRF2 with the downstream signaling pathway of the gonadal steroid receptor may provide specific indications to the mechanism of sexual dimorphism related to the effect of NRF2 on bone phenotype (43, 131–134).

## 4 NRF2 in Relation to the Development of the Human Skeleton

NRF2 in the femur were significantly reduced in osteoporosis patients (94). Multiple system disorders were caused by mutation in the activation of the human *NFE2L2* gene encoding NRF2, which impaired the inhibition of KEAP1 binding to NRF2, leading to marked dysregulation of gene expression and cytoplasmic redox imbalance. Although these patients exhibited no apparent malformed features, they developed a variety of abnormalities including developmental retardation, dysplasia, immunodeficiency, leukoencephalopathy and hypophomocysteinemia (135).

## 5 Conclusion

NRF2 and its resistance to oxidative stress are involved in bone metabolism. It is noteworthy that the compensatory increase of NRF2 may account for the self-protection of the bone injury caused by oxidative stress. It also be attributed to the induction of oxidative stress to the bone injury. The dose-dependent role of oxidative stress in bone cells has not been fully explored and should be further assessed in future studies.

However, it is still controversial in osteoblasts and during the osteogenesis of MSCs. The properties of NRF2 agonists have protective effects on the survival of osteogenic cells, including osteoblasts, osteocytes and stem cells. Most chemicals activate NRF2 to alleviate the damage of osteogenic differentiation and mineralization caused by high glucose, glucocorticoid, H_2_O_2_ and other stimuli. Activation of NRF2 directly inhibits osteoclast differentiation by resisting oxidative stress, whereas NRF2 indirectly promotes osteoclast differentiation by inhibiting the secretion of OPG from osteoblast, which does not show significant difference in the overall bone resorption in specific animal models. NRF2 agonists inhibit bone resorption by reducing ROS and blocking osteoclast differentiation. Although the effect of NRF2 inhibition and activation on animal bones are still controversial, most studies suggest that NRF2 is indispensable for the acquisition and maintenance of bone mass, as well as the protection of bone mass under various stress conditions, including acute loss of sex hormones caused by castration. Hyperactivation of NRF2 may cause damage to bone formation, while moderate activation of NRF2 promotes increased bone mass. In addition, the effects of NRF2 on the bone phenotypes were characterized by sexual dimorphism. NRF2 appears to be indispensable or harmless for bone mass in female mice, although conflicting results have been noted in male mice. We also need further studies on human skeleton and other system diseases and abnormalities.

To date, the role of NRF2 in bone metabolism has been investigated using global *Nrf2^–/–^* and *Keap1^–/–^* mice models. However, it may be difficult to exclude the influence of other tissues, such as endocrine glands on bone tissues and cells. To explore the specific role of NRF2 in different cell populations, more osteoclast-, osteoblast- and osteocyte-specific *Nrf2^–/–^*, *Keap1^–/–^* and *Keap1^+/–^* animal models should be established that aim to provide additional insight into the mechanism of NRF2 in bone homeostasis and metabolism.

In conclusion, the role of NRf2 in bone is complex and elusive by several factors, such as its range of activation, age, sex, the presence of various physiological and pathological conditions, as well as its interaction with certain transcription factors that maintain the normal physiological function of the bone tissue. The efficacy of NRF2-activated agents for bone protection and maintenance has been verified in a large number of *in vivo* and *in vitro* studies. More research on the role of NRF2 in bone metabolism will provide novel targets for the etiology and therapy of osteoporosis (136, 137).

## Author Contributions

JH and KY carried out the studies, participated in collecting data, drafted and prepared the manuscript. JA and NJ performed the statistical analysis and critically for important intellectual content. SF and XT considered the idea and overall structure of the article. All authors read and approved the final manuscript.

## Funding

This study was supported by the National Natural Science Foundation of China under grant no. 81370970, the Science and Technology Support Program of Gansu Province under grants no. 144FKCA075 and Gansu Province Endocrine Disease Clinical Medical Research Center (20JR10FA667).

## Conflict of Interest

The authors declare that the research was conducted in the absence of any commercial or financial relationships that could be construed as a potential conflict of interest.

## Publisher’s Note

All claims expressed in this article are solely those of the authors and do not necessarily represent those of their affiliated organizations, or those of the publisher, the editors and the reviewers. Any product that may be evaluated in this article, or claim that may be made by its manufacturer, is not guaranteed or endorsed by the publisher.
